# Skin-Related Adverse Reactions in Gadolinium-Based Contrast Agents-Induced Hypersensitivity: A Systematic Review of the Literature

**DOI:** 10.7759/cureus.80150

**Published:** 2025-03-06

**Authors:** Saraa A AL Jawad, Hussain A Abusreer, Ali A Ali, Alwaleed M Alamro

**Affiliations:** 1 Radiology/MRI, Omran General Hospital, Ahsa, SAU; 2 Radiology, Omran General Hospital, Alhasa, SAU; 3 Medicine, University of Khartoum, Khartoum, SDN; 4 Internal Medicine, Qassim University, Al-Qassim, SAU

**Keywords:** contrast-induced reactions, gadolinium-based contrast agents, hypersensitivity reactions, magnetic resonance imaging (mri), skin-related adverse reactions

## Abstract

Gadolinium-based contrast agents (GBCAs) have been used in clinical settings for several decades and in general they have established a good safety profile. Despite the use of chelating agents, some adverse reactions might occur when using these agents. This review aimed to assess the relationship between administered dose of Gadolinium-based contrast and the rate, duration, and severity of skin-related adverse reactions. Four electronic databases were searched using various relevant keywords. Duplicate records were removed, and the remaining records were retrieved and assessed for eligibility. The inclusion criteria were observational or experimental studies that analyzed the rate of immediate and/or delayed adverse reactions to GBCAs in magnetic resonance imaging. Data were extracted into a pre-designed spreadsheet. Ten studies were deemed eligible to be included in this review. A total of 691,007 GBCAs injections/patients were included in these studies. Six hundred seventy-nine skin-related adverse reactions were reported among the study participants, with a rate of 0.10%, urticaria was the most common (499 cases, 74.7%), while allergic dermatitis (three cases, 0.5%) and angioedema (four cases, 0.6%) were least frequent. In conclusion, the rate of these skin-related adverse reactions was low. The most commonly reported reactions were urticaria, rash, and pruritus. Most of these reported reactions were mild, no significant association was found in the volume of contrast agents and the risk for developing adverse reactions reported in the included studies.

## Introduction and background

Gadolinium-based contrast agents (GBCAs) have been used in clinical settings for several decades with a well-established good safety profile [1]. To promote the safety of gadolinium, all GBCAs incorporate a chelating agent, either linear or cyclic. This agent binds to gadolinium, improving its stability, solubility, and overall safety. Based on the molecular structure of the organic ligand, GBCAs are categorized as linear or macrocyclic. Additionally, they are classified as non-ionic or ionic depending on their net charge in solution [2].

Despite the use of chelating agents some adverse reactions (ARs) might occur. When GBCAs are administered at standard clinical doses (0.1-0.2 mmol/kg), the rate of ARs ranges from 0.07% to 2.4% [3]. These ARs can be local or systemic, local ARs, such as contrast extravasation, can occur with parenteral administration of GBCAs. However, due to the typically small volumes administered, these ARs rarely lead to serious harm. Patients with communication impairments, including children and individuals with debilitating conditions, as well as those undergoing multiple injections in the same vein or possessing fragile vasculature, are at increased risk [4].

Systemic side effects of contrast media can occur early (within 20 minutes) or late (after 20 minutes) and may be due to anaphylactoid reactions or the osmolarity and toxicity of the agent. Factors such as concentration, volume, and injection rate can also influence risk. Reactions range from mild to severe, with skin manifestations being the most common. While severe reactions are rare, accurate diagnosis is crucial as they can be life-threatening [5,6]. This review aimed to assess the rate of skin-related ARs in GBCAs and investigate their duration and severity and the impact of the administered GBCA volume on the risk of developing these ARs.

## Review

Methods 

This systematic review adhered to Preferred Reporting Items for Systematic reviews and Meta-Analyses (PRISMA) guidelines [7]. Four databases (PubMed, Scopus, Embase, and Cochrane Library) were searched using the following keywords: gadolinium, gadolinium-based contrast agents, GBCAs, skin lesions, dermatitis, urticaria, rash, erythema, exanthema, hypersensitivity reactions, allergic reactions, anaphylaxis, hypersensitivity, magnetic resonance imaging, MRI. Boolean operators, Medical Subject Headings (MeSH) terms, and other database-specific filters were employed. The search was limited to English-language studies on humans, excluding editorials, reviews, commentaries, case reports, conference proceedings, and abstracts.

Duplicate records were removed, and the titles and abstracts of the remaining records were screened to identify potentially eligible studies. Subsequently, full-text articles of the potentially eligible studies were retrieved and assessed for eligibility. The inclusion criteria were observational or experimental studies that analyzed the rate of immediate and/or delayed ARs to various classes of GBCAs in MRI. Immediate reactions were defined as allergic reactions that occur within 30 minutes of administration of the contrast agent, while delayed reactions manifest between one hour and seven days post-administration [8].

Data were extracted into a pre-designed spreadsheet. The extracted data included study design, country, number of participants and/or GBCA injections, duration of the study, type and dosage of GBCAs, number and types of skin-related ARs, information regarding the severity of ARs and their classification as either immediate or delayed. Records screening and data extraction were performed by all authors.

Results

**Figure 1:**
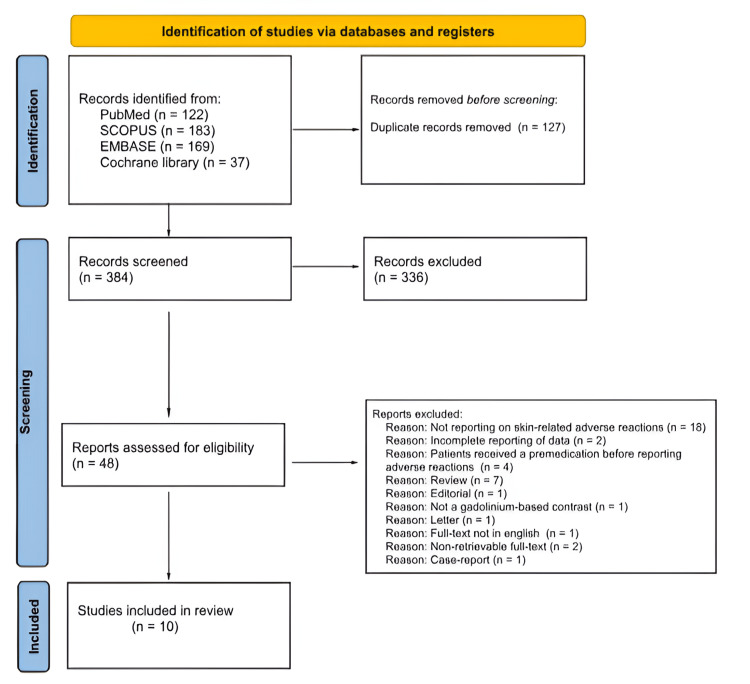
PRISMA flowchart of the study selection process. PRISMA: Preferred Reporting Items for Systematic reviews and Meta-Analyses

Following the title and abstract screening of 384 records, 48 were selected for full-text screening. Ten of them were deemed eligible to be included in this review. Figure 1 illustrates the study selection process. Basic characteristics of the included studies are shown in Table 1. Of the 10 included studies, only one of them was multinational involving 10 countries - four of the included studies were included in the US, while the rest were included in Canada, Germany, and China.

**Table 1:** Basic characteristics of the included studies. ARs: Adverse Reaction, GBCA: Gadolinium-Based Contrast Agents

Study	Country	Design	Duration	Number of patients/ injections	Contrast media	Reported skin ARs
McDonald 2019 [9]	The United States of America	Retrospective study design	8 years	281,945 GBCA injections	Gadodiamide, Gadobutrol, Gadobenate dimeglumine, Gadoterate meglumine	Urticaria
Walker 2019 [10]	Canada	Retrospective cohort study	8 years and 8 months	551 patients	Gadobutrol	Urticaria, erythema, pruritus, cutaneous edema
Forbes-Amrhein 2018 [11]	The United States of America	Retrospective cohort study	8 years and 1 month	32,365 GBCA injections	Gadopentetate dimeglumine, Gadoterate meglumine, Gadoxetate disodium, Gadofosveset trisodium	Urticaria, cutaneous edema, pruritus, diffuse erythema
Glutig 2018 [12]	Germany	Prospective, study design	2 years and 8 months	3,710 patients	Gadobutrol	Allergic dermatitis, erythema, allergic pruritus, urticaria
Power 2016 [13]	Canada	Retrospective cohort study	4 years	30,373 GBCA injections	Gadobutrol	Urticaria, rash, pruritus, limited erythema
Soyer 2016 [14]	Multinational (10 countries)	Prospective cohort study	4 years and 6 months	35,474 patients	Gadoterate meglumine	Urticaria, angioedema, pruritus, rash, allergic dermatitis, erythema
Aran 2015 [15]	The United States of America	Retrospective study design	7 years	194,400 GBCA injections	Gadopentetate dimeglumine, gadobenate dimeglumine, gadoxetate disodium, gadofosveset trisodium	Erythema, pruritus, rash, cutaneous edema
Dillman 2007 [16]	The United States of America	Retrospective study design	5 years	78,353 GBCA injections	Gadopentetate dimeglumine, Gadobenate dimeglumine, Gadodiamide	Urticaria, rash, cutaneous edema
Herborn 2007 [17]	Germany	Retrospective study design	1 year and 9 months	24,308 patients	Gadoterate meglumine	Injection site pain, pruritus, urticaria
Li 2006 [18]	China	Retrospective study design	4 years and 11 months	9,528 patients	Gadoterate meglumine, Gadodiamide, Gadopentetate dimeglumine	Urticaria, rash

Regarding the study design of the included studies, five of them followed a retrospective cross-sectional study design. The duration of the studies ranged between one and eight years. The studies included a total of 691,007 GBCA injections/patients. The ARs were investigated in different GBCAs including Gadodiamide, Gadobutrol, Gadobenate dimeglumine, Gadoterate meglumine, Gadopentetate dimeglumine, Gadoterate meglumine, Gadoxetate disodium, and Gadofosveset trisodium. The studies investigated a variety of skin-related ARs, the most reported of which was urticaria in all of the studies.

A total of 679 skin-related ARs were reported among the study participants, with a rate of 0.10% out of a total of 691,007 GBCA injections/patients. The most commonly reported AR was urticaria with 499 reactions and 74.7% of total skin-related reported ARs. The least reported ARs were allergic dermatitis and angioedema which composed three (0.5%) and four (0.6%) of total skin-related reported ARs, respectively. Table 2 demonstrates the reported skin-related ARs in the included studies.

**Table 2:** Reported skin-related adverse reactions in the included studies.

Skin-related adverse reactions	Frequency (percentage)
Urticaria	499 (74.7)
Rash	85 (12.7)
Pruritus	38 (5.7)
Erythema	30 (4.5)
Cutaneous edema	15 (2.3)
Injection site pain	5 (0.8)
Angioedema	4 (0.6)
Allergic dermatitis	3 (0.5)
Total	679 (100)

Information about the dodge of GBCAs dosage was reported in seven of the included studies. The average dose administered was 0.1 mmol/kg. No significant difference was found in the volume of the administered GBCAs between patients who had ARs and those who did not. Power et al.’s study was the only study that arranged the reported ARs as immediate and delayed, most of the reported ARs 100 (85.5%) had an immediate emergence [13]. 

Five of the included studies reported on the severity of skin-related ARs. in all of the studies skin-related ARs were mostly mild. In Walker et al.’s study all of the reported skin-related ARs were mild [10]. The same was also reported for Dillman et al. and Li et al. [16,18]. Regarding Power et al.'s study, most of them were mild with exception of four ARs which were moderate in nature [13]. Aran et al.’s study also reported that 87.7% of ARs were mild, 10.5% were moderate, and 1.8% were severe [15].

Discussion

The precise mechanisms underlying contrast media-induced ARs remain unclear and likely involve multiple factors. While type-I hypersensitivity is considered the primary mechanism, additional pathways may contribute. These include histamine release from mast cells and basophils, as well as complement system activation. Moreover, it has been suggested that the osmolarity and chemical structure of contrast media can directly affect cells, leading to membrane alterations and subsequent allergic responses [19]. In general, non-iodinated contrast agents such as GBCAs are associated with a lower risk of ARs compared to iodinated contrast media [20], which is evident in our study since the overall rate of skin-related ARs was 0.10%, compared to the reported rate of 1.15% for iodinated contrast media in a study conducted by Ho et al. [21].

In this systematic review all of the studies that assessed the association between the volume of the administered GBCAs and the risk for ARs found no significant association, this was similar to what has been found in previous study [22]. However, Jung et al. found a slight increase in the rate of severity of ARs by 9.8% in the population who had higher volume of administered GBCAs [23]. Although we did not compare the rates of ARs between different types of GBCAs, it has been stated that nonionic GBCAs had the lowest rates of ARs compared to the others [24,25]. Ionic contrast agents dissociate into positively and negatively charged ions upon intravenous administration. This process leads to a doubling of the particle count in solution, resulting in increased osmolality and viscosity. These factors may contribute to a higher incidence of ARs [26,27].

Nonionic GBCAs might have a lower risk for ARs however that have been linked with an increased risk for nephrogenic systemic fibrosis [28]. Given that the risk of nephrogenic systemic fibrosis can be mitigated by pre-assessing the renal function, nonionic GBCAs may be a suitable choice for patients with normal renal function who exhibit a higher risk of allergic reactions. Such patients may include individuals with asthma, severe allergies, or a history of ARs to other GBCAs [29].

For patients who had previous reactions, the practice of transitioning to new GBCA has been reported to be useful. The American College of Radiology and the European Society of Urogenital Radiology suggest that using a different GBCA may reduce the risk of subsequent reactions [30]. Patients with a previous severe reaction to a contrast agent have a significantly elevated risk of subsequent reactions, approximately five to six times greater [31]. Similarly, individuals with a history of allergies or atopic conditions like asthma, dermatitis, and urticaria, exhibit a three- to six-fold increased risk of severe reactions to contrast media [32].

For patients considered at risk, premedication may be considered, although its efficacy in preventing acute allergic-like reactions remains unproven [33]. Glucocorticoids exert anti-inflammatory effects by binding and inhibiting pro-inflammatory genes, such as interleukin-1 [34]. Additionally, these agents impair neutrophil migration, macrophage function, and mast cell cytokine production and degranulation, leading to a weakened innate immune response, which is typically activated rapidly and independently of preformed antibodies. Oral administration of premedication is preferred and should be administered at least six hours prior to contrast agent injection [35].

One of the limitations of this review is that most of the included studies used a retrospective design. This approach may have introduced selection bias and relied on records to ensure data accuracy. Additionally, as not the same GBCAs were utilized by all studies, variations in event reporting practices across individual studies may have influenced the review results.

## Conclusions

This review aimed to assess skin-related ARs of GBCAs. We found the rate of these reactions was low with a rate of 0.10%. The most commonly reported reactions were urticaria, rash, and pruritus. Most of these reported reactions were mild in nature, and no significant association was found in the volume of contrast agents and the risk for developing ARs reported in the included studies.
